# Optimizing Film Companies' Marketing Strategy Using Blockchain and Recurrent Neural Network Model

**DOI:** 10.1155/2022/4139074

**Published:** 2022-09-09

**Authors:** Yahui Yu, Jie Liu

**Affiliations:** ^1^Communication University of Shanxi, Jinzhong City 030619, China; ^2^Shanxi Vocational College of Art, Taiyuan City 030001, China

## Abstract

With the growing scale of the domestic film industry, the output of domestic films is also increasing. The design and execution problems in ticket marketing strategies adversely affect the revenue of domestic films. This paper aims to propose solutions to optimize film companies' marketing strategies by analyzing the marketing environment and current situation to increase the income of domestic films. Firstly, the current situation of BJ's marketing is analyzed, and the main problems are clarified. Secondly, the necessity and feasibility of applying blockchain technology to the operation and management of film companies are introduced. New marketing strategies and safeguards have been developed by analyzing the target market and optimizing the program. A quantitative method is used to predict a new movie's box office in its premiere month. The proposed method introduces three factors affecting the box office: new film positioning, film marketing, and film prerating, as input variables. Besides, a Recurrent Neural Network (RNN) is implemented to predict the monthly box office of the premiere of new films. The results show that the predicted monthly box office of the movie adopting the optimized marketing strategy is 1,451,718.6 CNY, which is smaller than the target box office of 1414029.8. The Mean Absolute Error (MAE) is only 0.026. The proposed model's Root Mean Square Error (RMSE) is 0.45 for the long-term prediction of a single movie price. The MAE is 0.106, and the accuracy is 0.80. The model proposed beats the unimproved Long Short-Term Memory (LSTM) model and the Autoregressive Moving Average (ARMA) model. This paper provides a reference for optimizing the film company's marketing system.

## 1. Introduction

China's tertiary industry has developed rapidly, and residents' disposable income has continued to increase. How to further satisfy the spiritual life of the Chinese has become the adjustment direction of China's industrial policy at present and in the future. The satisfaction of spiritual life requires cultural connotations with the characteristics of the times. It is vital to develop and enhance China's cultural industry effectively. Meanwhile, it has become a key task of the cultural industry to deeply explore the excellent cultural connotation of China and visualize it as an easy-to-understand way to meet the Chinese spiritual and cultural needs. This is also the core content of a sustainable development-oriented cultural industry [1, 2]. Indeed, the film industry plays a central role in the cultural industry development as an important pillar industry in China. It provides rich content and creative resources for the entire cultural industry. Therefore, the film industry plays a pivotal role in the entire cultural industry. China's film industry has seen decades of steady development, with a double-digit growth rate in economic income increase yearly. The Chinese film industry has laid a solid foundation for the healthy and sustainable development of the cultural industry. The Chinese film market is still in an upward cycle, and the market potential is still large. However, on the one hand, many high-quality foreign films have been introduced into China with the globalized market. It will certainly impact the domestic film industry [3, 4], increasing market competition's intensity. On the other hand, the application scope becomes extensive as film production technology matures. Especially e-commerce giants like Alibaba and Amazon extensively use big data technology to collect consumers' individual characteristics and consumption habits. Based on this, they recommend accurate products and services, promoting marketing. It is now urgent for film companies to introduce big data analysis techniques and construct precision marketing systems over the previous marketing model. By doing so, film companies will see better marketing effects, reduce costs, and push products & services to potential customers timely, effectively, and accurately. Then, the audience's spiritual and cultural needs are met to help film companies gain a competitive advantage in the fierce market [5, 6].

Ruo-Yu and University [7] pointed out that the Chinese people's energy and financial investment in infants and young children continued to increase with the improvement of national quality and the economy. The maternal and infant products market was in a special period of sustained prosperity and development. This era had given mother and baby product companies opportunities for development. There were many problems in the marketing strategy of China's mother and baby products market. The maternal and infant market problems and the corresponding optimization strategies were discussed. Wu [8] argued that new media advertising marketing was fundamentally different from the original advertising marketing in the context of the “Internet +” era. For the above problems, corresponding strategies were proposed. Specifically, they included enhancing the dominance of communicators, optimizing the internal industrial structure of new media, expanding advertising channels, and determining the cultural characteristics of advertising audiences. The proposed strategy could optimize new media marketing methods and the information dissemination ability of advertising companies. These strategies also improved economic efficiency and China's new media advertising industry. Cheng et al. [9] observed that consumption upgraded, and consumers' demands were diversified in the context of retail. The development of traditional e-commerce enterprises has encountered bottlenecks. Freshippo was a pioneer in the new retail of fresh food e-commerce. The experiential marketing strategy of Freshippo was analyzed based on its development overview, focusing on five aspects: sensory experience, emotional experience, thinking experience, action experience, and relationship experience. Finally, some revelations were presented.

This paper reviews relevant literature and believes that obtaining consumers' individual characteristics and preferences through various channels in constructing the precision marketing system is necessary. Then, relevant analysis tools are used to analyze the obtained data, and the internal correlation between the information provided by consumers and the product information can be effectively mined. Based on this, consumers are classified and identified to push targeted products accurately. A comprehensive consumer-company communication platform is built to meet consumer needs through marketing efforts. It quickly, accurately, and appropriately stimulates consumers' purchase intention and reduces marketing costs, and improves marketing efficiency. Furthermore, it is found that foreign countries start early in precision marketing and have formed relatively rich research results. Although China starts late in precision marketing, it has also made remarkable achievements in research fields and content. Especially in the fast-moving consumer goods and online consumer industries, it is catching up quickly and achieving obvious results. Therefore, it is important to apply the theory of precision marketing to the film industry to construct a precision marketing plan for the Chinese film industry and film companies. Precision marketing requires film companies to pay enough attention to their marketing concepts and invest money and energy to dig deep into moviegoers' individual characteristics and viewing experiences using big data. Moreover, there are big differences between Eastern and Western consumers regarding consumption concepts and preferences. Therefore, whether foreign research results can directly guide domestic practice needs further verification and discussion. For domestic enterprises, domestic consumers' consumption habits and concepts should be fully considered in terms of individual characteristics and consumer preferences. Enterprises should obtain consumers' consumption information truly and effectively and provide comprehensive, objective, and real information data to construct a precision marketing system.

The research significance covers two aspects: theoretical and practical significance. In terms of theoretical significance, this paper is based on precision marketing theory and formulates a scientific and reasonable marketing model for the film industry through extensive research and in-depth analysis, especially in the film promotion stage. This improves the accuracy and timeliness of video publicity to expand the scope of application of the theory. In terms of practical significance, this paper introduces the theory of precision marketing into the marketing system of the film industry. It improves the marketing model of the film industry and subverts the traditional marketing concept. It also further improves the film's marketing efficiency and increases the film's viewing volume, solving the current challenges faced by the film industry in the market and production. This provides new solutions for Chinese film marketing.

## 2. Marketing Status of BJ Film Company

BJ Film Company is a state-owned listed film company established in 2005 and listed on the Growth Enterprise Market (GEM) in 2016. Under the general trend of the film market economy, in the early days of its establishment, BJ Film Company basically adopted the method of participating in the joint distribution of films. This method can accumulate experience and reduce the risk of independent production. After a period of experience accumulation, BJ Films quickly started independent production, achieving good results in sales and word of mouth. As the domestic film market invests heavily in film marketing, BJ Film Company is still in the initial stage of development. Hence, its marketing effort is far from enough.

Generally, film marketing strategy is divided into product strategy, channel and price strategy, and communication strategy. BJ Films is committed to diversification and quality from the product strategy perspective. In addition, the customer market has been preliminarily segmented, and the different customer-tailored film marketing has expanded the company's influence. Meanwhile, the company considers the regional differences and income distributions in pricing tickets to increase its competitive advantage in the market. Over the years, BJ Films has been aware of the fierce competition in the film industry. In the film market, the content and quality of films are still the most important competitiveness, but to win in the industry, the later release of the film and marketing processes are also increasingly significant. Therefore, BJ Films hopes to change the existing marketing model.

The company's film business income mainly comes from film production and distribution, theater distribution and screening, and movie studio sales. From the perspective of film production and distribution, BJ Films increased its investment in film distribution and production last year. It adheres to the development strategy of the film industry chain, the dual drive of “content + channel,” and optimizing the industrial structure. The operating cost is nearly 180 million CNY, but the operating income is only 207 million CNY, and the expenditure is not proportional to the income. From the perspective of theater distribution and screening, BJ Films still has many marketing and film distribution problems. The company's film business segment has an income of 1.65 billion CNY, of which the theater distribution and screening business income is 1.379 billion CNY, accounting for nearly 84% of the revenue of the film business segment, and the main source of revenue is still relying on theater distribution and cinema screening business. This also means that BJ's revenue in the business segment relies too heavily on theaters, and the revenue structure is still unbalanced and unhealthy, demanding enrichment. Further, although BJ Films has produced and released a number of films, not every film has been released in theaters. Among the films released in theaters, there are many works with a good reputation, but the box office is unsatisfactory. It may be mainly due to BJ Films' problems with marketing and distribution.

The marketing problems of BJ Films can be summarized into three points: First, the positioning of the film is not clear, the main customer group is not clear, and they blindly follow the trend and enter the market. For example, comedies and youth dramas are young man's favorite when socializing, such as dating. Thus, these films have to be exhibited on public holidays or after work when more youngers are available on the market [10]. Second, the publicity and distribution malfunctions without a detailed analysis of the market demand and the absence of Internet technology. The selected materials and visual image themes are not divided and cannot target specific consumer groups. Many publicity materials are filmed following the trend, and audience groups are not selected, leading to a poor box office [11]. The content lacks innovation, and the production is selected purely based on the subjective perception of the enterprise. Third, the marketing and promotion methods are single, and the new media and other means are not fully utilized for promotion. It is necessary to use various methods, such as the Internet and other advanced technologies, to promote and market films to increase their popularity and the box office [12].

## 3. Optimization of Marketing Strategy of Film Companies

### 3.1. Blockchain-Based Marketing Strategies

The blockchain is a distributed digital ledger that records information and data. The ledger is stored among multiple participants in a peer-to-peer network. Participants can use encrypted signatures to add new transactions to the existing transaction chain, forming a secure, continuous, immutable, and chained data structure. From the perspective of data, immutability is a major feature of blockchain [13, 14]. Therefore, the characteristics of blockchain include distribution, multinode consensus, openness, transparency, and immutability. Hence, the blockchain has many technical advantages in information sharing, reducing the cost, manual work, and the possibility of manual errors. Large-scale applications make blockchain a breakthrough digital technology after the Internet [15].

**Figure 1:**
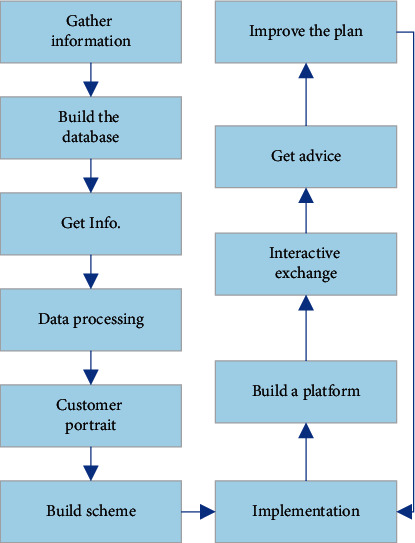
The process of the marketing execution.

The marketing of BJ Films should start from data acquisition and explore moviegoers' individual characteristics and viewing needs. The internal logic and related information must be mined to formulate precise marketing strategies. Also, the marketing strategy should strengthen the interaction with moviegoers and respond appropriately to audience feedback [16]. The specific process is shown in Figure 1.

The marketing execution can follow four steps.

1. Help marketing through high-quality official accounts. Film production and distribution companies can recruit professional personnel to establish and maintain official accounts [17]. WeChat official accounts can implant movies into public accounts in various ways, which can also greatly promote communication. This method can also promote the creative space of communicators and convert the original video or picture-based single communication mode into a combined marketing mode [18]. Companies must pay attention to the quality of the disseminated content to disseminate and recommend movies on the official account. Specific efforts can include the text's content expression, the picture's design, the video's duration, and the quality of the edited content. The text content can greatly influence fans' judgment of the quality of the official account. However, suppose the Weibo promotions and official advertising overlap too much. The marketing effect will be discounted or even negatively affected in that case. This is where innovative ideas come to play. The images and videos give fans a general idea of what the film is about through a brief introduction, increasing their desire to watch it.
2. Position the user thinking through the publicity and distribution of materials. The positioning of differentiated marketing can be used as the starting point to form appropriate themes and carry out targeted communication over the Internet. After the content and audience positioning, the user attribution platform is defined, such as Weibo, WeChat, and Zhihu. At the same time, based on new technologies and viewing data, targeted promotional materials will also be launched online and offline. Communicators should establish exclusive “databases” for different consumer groups by positioning differentiated marketing and audiences. They should promote two-way communication in film marketing and implement targeted marketing strategies following sufficient user information [19, 20].
3. Use a short video to publicize the film. As of June 2020, the number of short video users in China reached 818 million, surpassing comprehensive video and becoming the second-largest application after instant messaging [21]. With the solidification of media output channels in recent years, homogenization has become a prominent problem in film and television marketing. From the user's point of view, the post-90s and post-00s are no longer satisfied with a single film and television promotional video or tidbits but pursue personalization, and large traffic is no longer the panacea to open the market. For example, Tik Tok's short video marketing has gotten rid of the one-way output in long-term film and television marketing. It now outputs marketing videos in a form that users like to hear. The natural combination of film and television works and the popular content of Tik Tok can easily shorten the distance between the works and the audience. Letting users become marketing self-media and achieve secondary communication in their respective circles can have a good publicity effect.
4. Cultivate the user community of new social media. Interpersonal communication is the most common and important way to transmit information, featuring timely feedback and individual interaction [22]. In today's society, interpersonal communication has risen to group communication, which has the characteristics of similar attributes and common goals. The emergence of new media has created conditions for medium and low-cost films to cultivate audience communities and promote their publicity. With the help of community content mechanisms such as comments and recommendations, the frequency of social interaction with the audience can be increased. The content stickiness of users in the target community can also be enhanced, forming a community force that spontaneously participates in publicity.

The process of publicity and distribution after the film is released can be divided into three stages: the early, the middle, and the later. In the early stage, posters, videos, and other materials are used in marketing, and Weibo-based materials are launched to drive other social media. In the mid-term, the exposure of topics will be increased through content marketing, music marketing, brand linkage marketing, and Key Opinion Leader (KOL) [23]. The later marketing mainly includes maintaining film word of mouth and developing related brand cooperation products. Related movie products can be developed for fans to encourage sales, such as books, actors' water cups, canvas bags, posters, and postcards. This helps increase movie revenue.

**Figure 2:**
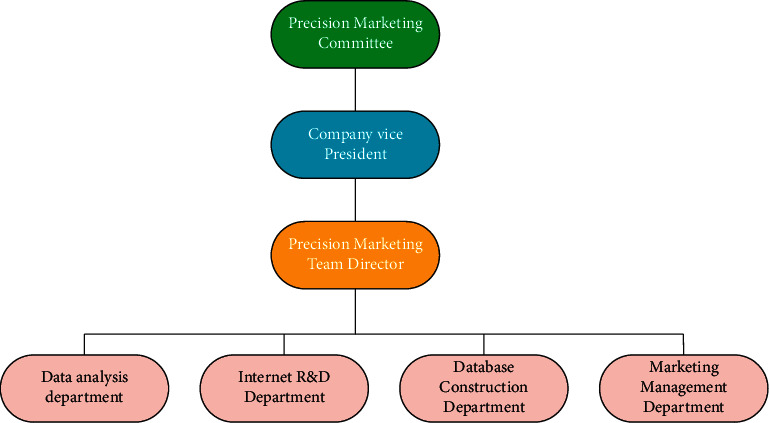
The organizational structure of the marketing team of BJ Films.

Further, corresponding safeguards must be formulated to ensure that the new marketing strategy can be implemented as planned. For BJ Films, the core of its precision marketing system is professionals with strong precision marketing and data analysis capabilities [24]. To this end, BJ Films must make adjustments at the organizational design level to ensure the precision marketing system's effective construction and smooth implementation. The organizational structure of the marketing team of BJ Films is shown in Figure 2.

While building a new marketing strategy, BJ Films should also speed up constructing the relevant security system. The company should appropriately approach the marketing department regarding resources and policies, allocate resources reasonably, optimize the management model, and establish an incentive-compatible reward and punishment system [25]. The work responsibility and subjective motivation of the marketing department should also be stimulated to ensure that the marketing strategy guarantees the company's development [26].

### 3.2. Marketing Model Based on Recurrent Neural Network (RNN)

**Figure 3:**
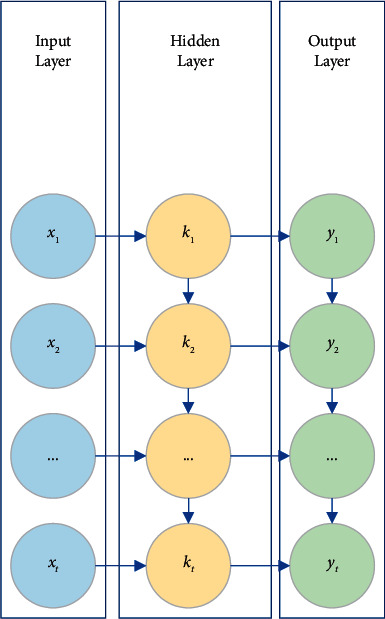
The network structure of the RNN.

This section introduces RNN to establish a marketing model to predict the monthly box office of the film's premiere using the new marketing strategy. The RNN will memorize the previous information and apply it to calculate the current output. The network structure of the RNN is shown in Figure 3.

Assuming that at time t, the input is x_t_, the state of the hidden layer is k_t_, and the functional relationship is shown in the following equations:

(1) $$\begin{array}{c} k_{t}=f\left(Uk_{t-1}+Wx_{t}+b\right)\text{,} \end{array}$$

(2) $$\begin{array}{c} y_{t}=softmax\left(V_{t}\right)\text{.} \end{array}$$

**Figure 4:**
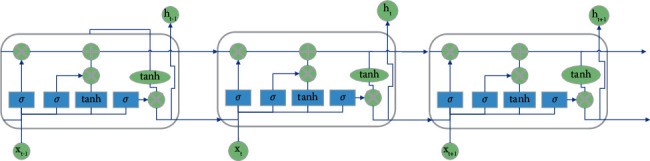
The network structure of LSTM.

In equations (1) and (2), U, W, and V are all weights, b is the threshold, and softmax is the activation function. In order to traffic congestion and reduce traffic pressure, the latest research adopts the Bi Long-Short Term Memory (BiLSTM) algorithm to predict traffic flow. It establishes the algorithm network of urban road short-term traffic state based on BiLSTM and optimizes the internal storage unit structure of the network. Thereby, a high-quality prediction model is built. It is found that the prediction results of LSTM and BiLSTM are consistent with the actual traffic flow trend [27]. Based on its superior prediction characteristics, this paper also takes LSTM as one of the research methods. Theoretically, RNN can process sequence data of any length. However, due to the vanishing or exploding of the gradient of RNN, it can only learn short-period dependencies. In practice, to reduce complexity, it is often assumed that the current state is only related to a few previous states [28]. Therefore, an improved method of RNN, Long-Short Term Memory (LSTM), can be used to solve the gradient problem of simple RNN. The LSTM model refers to a set of memory units. At time t, memory unit c_t_ records all historical information up to the current time and is controlled by the input gate i_t_, forget gate f_t_, and output gate o_t_. The element value of the three gates is between (0,1), and the input gate i_t_ controls the amount of new information added to each memory unit. The forget gate f_t_ controls the amount of forgotten information in each memory unit. The output gate o_t_ controls the number of output information of each memory unit [29, 30]. The network structure of LSTM is shown in Figure 4.

In Figure 4, the output vector h_t−1_ of the previous time stamp and the input x_t_ of the current time stamp pass through the activation function tanh to obtain a new output vector h_t_. *σ* is the sigmoid activation function. Based on this chain, LSTM improves the interior of the module and uses three sigmoid layers and a gate composed of point-by-point multiplication operations to strengthen the information control ability [31]. The tanh activation function mainly processes the data for the state and output functions. The input gate controls the input of the output information of the upper layer unit to the unit information of this layer and retains the past information of the sequence. The calculation of each threshold layer is shown in equations (3) to (5).

(3) $$\begin{array}{c} f_{t}=\sigma \left(W_{f}\cdot \left[h_{t-1},x_{t}\right]+b_{f}\right)\text{,}\\ i_{t}=\sigma \left(W_{i}\cdot \left[h_{t-1},x_{t}\right]+b_{i}\right)\text{,}\\ o_{t}=\sigma \left(W_{o}\cdot \left[h_{t-1},x_{t}\right]+b_{o}\right)\text{.} \end{array}$$

In equations (3) to (5), W is the weight of the threshold layer, and b is the offset of the threshold layer [32]. After the update of each threshold layer is completed, the memory unit c_t_ is updated again. The calculation is shown in the following equation:

(4) $$\begin{array}{c} c_{t}=f_{t}*c_{t-1}+i_{t}*\;\tanh\;\left(W_{c}\cdot \left[h_{t-1},x_{t}\right]+b_{c}\right)\text{.} \end{array}$$

In equation (4), W is the weight of the threshold layer, *b* is the offset of the threshold layer, the NN output weight q_t_ of the internal memory is controlled by the output gate, and the activated unit state is output to the next neural network layer and chain units [33]. The specific calculation is shown in the following equation:

(5) $$\begin{array}{c} q_{t}=o_{t}*\;\tanh\left(c_{t}\right)\text{.} \end{array}$$

The sigmoid activation function outputs the memory state of the network. When a piece of data is inputted, the sigmoid activation function will compare it to [0, 1]. Here, 0 means that no amount is allowed to pass through, and 1 represents that any amount can pass [34]. Suppose the output value is within the specified range. In that case, the current layer's output value and calculation result are multiplied by a matrix. The result is input to the lower layer map, with a real-number range of [0, 1]. The function value represents the probability of a positive class [35]. Its expression is shown in the following equation:

(6) $$\begin{array}{c} f\left(z\right)=\frac{1}{1+e^{-z}}\text{.} \end{array}$$

The tanh activation function is different from the sigmoid activation function, which can map the real-number domain to the range of [−1, 1]. When the input is 0, the output is also 0 [36]. The expression is shown in the following equation:

(7) $$\begin{array}{c} f{\left(x\right)}_{\tanh}=\frac{e^{x}-e^{-x}}{e^{x}+e^{-x}}\text{.} \end{array}$$

In the training stage, LSTM learns the weights and offsets of each threshold layer from the past information. In the stage of real-time prediction, the trained model is used to operate the input data to obtain the predicted value of the time series. It thereby improves the efficiency of past mining information and shortens the training time [37].

**Figure 5:**
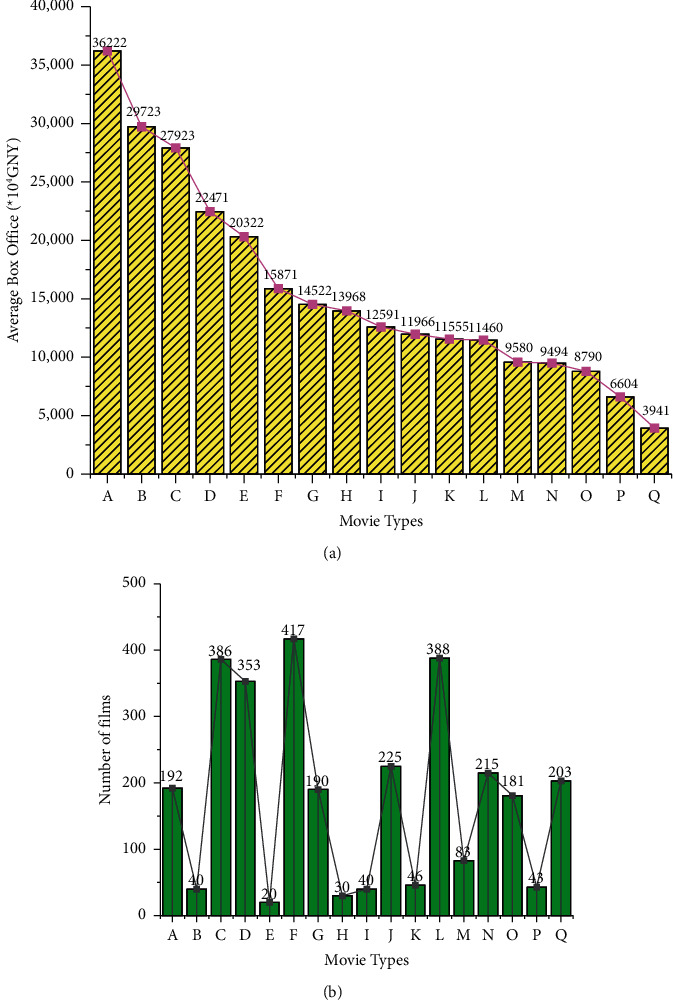
Descriptive statistics of the type of films. ((a) is the average of the total box office of each type of film, (b) is the number of each type of film).

Many factors affect the box office of a film. These factors include the information that the producers and distributors use to increase consumers' objective perception of movie products, such as movie type, brand value, and star effect. Also, audiences' opinions are also considered to increase consumers' subjective perception of movie products, such as film ratings [38]. This paper mainly studies the influence of film companies' marketing strategies on the box office of films. Thus, it only selects the type of films, the rating of films, and the marketing of films as the main factors affecting the box office. Of these, the type of films is divided into 17 according to the definition, denoted as variables A-Q. The rating of films includes one variable, R. The marketing of films includes three variables: audience demand, marketing methods, and publicity efforts, which are S, T, and U, respectively [39]. Altogether 21 variables are input into the LSTM model. The sample of the model selects 1,967 commercial films that were publicly released nationwide from 2016 to 2021. The average total box office and the number of each type of film are grouped and counted. The results are shown in Figure 5.

The meanings of A-Q in Figure 5 are Fantasy Movies, War Movies, Action Movies, Adventure Movies, Sports Movie, Comedy, Crime Movie, Musical, Historical Movie, Drama Movie, Western Movies, Romantic Movie, Ethical Films, Cartoons, Inference Movie, Documentaries, and Horror Movie.

**Figure 6:**
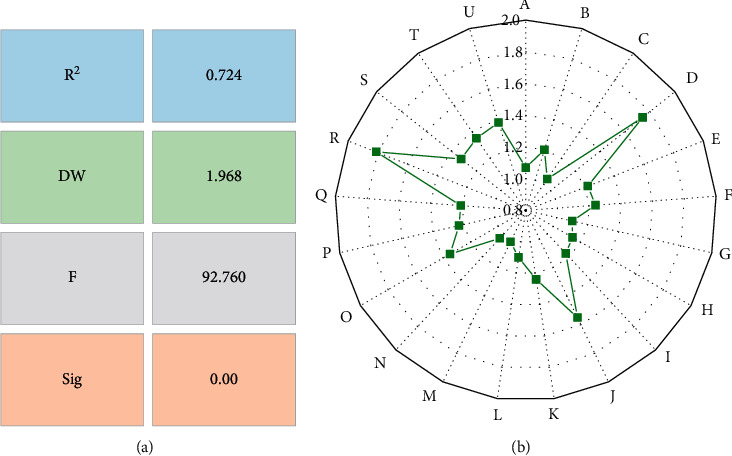
Summary of model indicators. (a) The model indicators; (b) The VIF value of each variable.

Large differences exist in the value of the film's box office. Thus, to eliminate the influence of heteroscedasticity in the model, the logarithm of the film's box office is removed. The logarithm with base *e* is selected. The obtained model indicators are shown in Figure 6.

In Figure 6, after the model is established, the *R*^2^ value is 0.614, indicating that the model has a good fit. The variance inflation factor (VIF) is less than 10, indicating no collinearity. The constant term of the model is tiny, which can be a box office prediction for any film.

### 3.3. Experimental Verification

**Figure 7:**
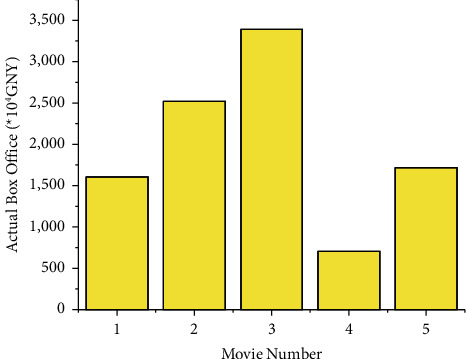
The box office of the five released films.

The experimental verification is divided into two parts. Firstly, the box office prediction model based on the RNN is used to predict the five films that have been released. The marketing variables are input variables using nonoptimized marketing strategies. The predicted box office is compared with the actual box office, proving the model's applicability and accuracy. The box office of the five released films is shown in Figure 7.

**Figure 8:**
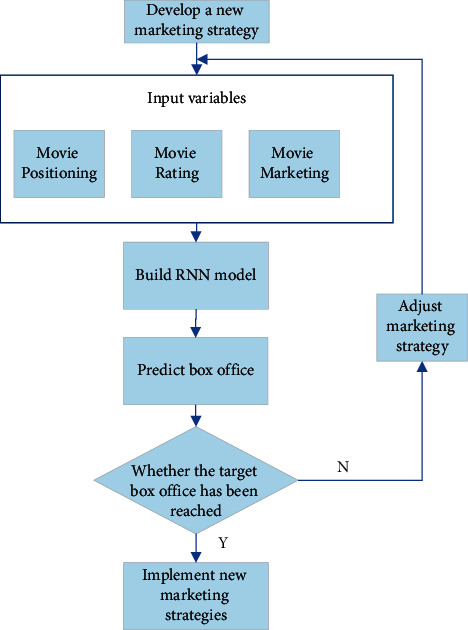
Adjusting marketing strategies by predicting box office.

Secondly, the optimized marketing strategy is used as the input variable to predict a film that will be released. The predicted box office is compared with the target box office. Suppose the predicted box office reaches the target box office. In that case, the film's marketing strategy formulation and implementation are relatively successful. Suppose the predicted box office does not reach the target box office. In that case, the film's marketing strategy will be adjusted in time. The specific idea is shown in Figure 8.

The theater box office refers to the sales revenue that theaters receive from selling movie tickets when a movie is released. Regarding the current state of Chinese theater operations, theater box office revenue accounts for about 80% or more of the total theater revenue. Therefore, predicting the box office revenue of theaters is important for the feasibility analysis of theater investment and business strategies. The theater's seat number will be overestimated by “total seats × average actual screenings” when the screening count with minimum seats is less than the average. Conversely, the theater's seat number will be underestimated by “total seats × average actual screenings” when the screening count with minimum seats is more than the average. Therefore, the influencing factors of “average actual screenings” and “total seats × average actual screenings” should be considered in predicting the box office revenue. Then, it chooses a reasonable calculation value to improve the accuracy of the prediction result.

## 4. Results and Analysis

### 4.1. Validation of Model Suitability

**Figure 9:**
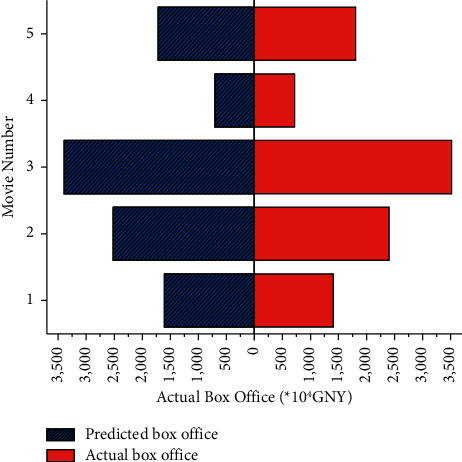
Comparison of actual box office and predicted box office of released films.

The five films that have already been released are predicted by the proposed RNN-based box office prediction model. The marketing variables are input variables of nonoptimized marketing strategies used before the release of the five films. The predicted monthly box office and the actual value are compared in Figure 9.

In Figure 9, the predicted results are consistent with the actual results. The obtained errors are taken as positive values and then averaged. Finally, the average relative error of the RNN is 7.7%, and the prediction accuracy reaches 92.3%. The result proves that the proposed box office prediction model based on RNN has certain accuracy and applicability. The marketing strategy of film companies can be optimized through this model.

### 4.2. Feasibility Verification of Adjusting Marketing Strategy by Predicting Box Office

**Figure 10:**
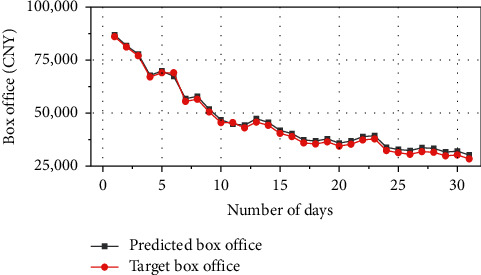
Comparison of predicted box office and target box office of unreleased films.

The proposed box-office prediction model based on RNN is used to predict a film that has not yet been released. The marketing variables adopt the proposed marketing strategy based on blockchain technology as input variables. The predicted and the target monthly box offices are compared in Figure 10.

In Figure 10, for this unreleased film, the predicted monthly box office is 1,451,718.6 CNY, reaching the target monthly box office of 1,414,029.8 CNY. The relative error is only 2.6%, indicating that the proposed marketing strategy has met the marketing needs of this film. It is feasible to adjust the marketing strategy by predicting the box office.

### 4.3. Index Prediction Results

**Figure 11:**
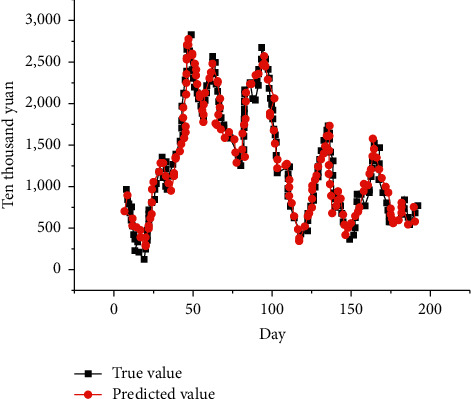
Prediction results of the movie's comprehensive box office.

Figure 11 shows the prediction results of the movie's comprehensive box office. The black line is the actual value, and the red line is the predicted value.

From Figure 11, the prediction value of the model constructed here is close to the true value of the movie's comprehensive box office, with a small deviation. The model proposed has little difference in the fitting curves of the prediction results of different indices. Therefore, the model has generality in predicting the comprehensive box office of movies.

### 4.4. Comparison of Movie Ticket Price Prediction and Algorithms

**Figure 12:**
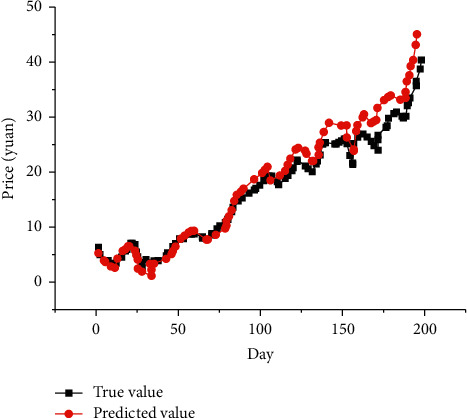
Ticket prediction results.

Figure 12 reveals the results of the long-term prediction of movie ticket prices by the improved network. In Figure 12, the abscissa is time, and the ordinate is the ticket price. The black line is the actual value, and the red line is the predicted value.

From Figure 12, the network's predicted ticket price in the first 100 days fits the true price perfectly. There is a gap between the predicted and the actual ticket prices in the interval of 100 days to 200 days, but it does not affect the short-term prediction.  Table 1 shows the performance of the network.

The proposed model's prediction errors are compared with the unimproved LSTM model and the Autoregressive Moving Average (ARMA) model. The comparison results are shown in Table 2.

From Table 1, the proposed model here has an RMSE of 0.45 for the long-term prediction of a single movie ticket price, an MAE of 0.106, and an accuracy of 0.80. From Table 2, the model proposed here has the smallest error and the best performance. This model can well describe the price trend of the movie market.

## 5. Precise Marketing Strategies Based on Multichannel

BJ Film Company analyzes the individual characteristics data, movie preferences, and other information of the movie viewing group to form a movie viewing group's segmentation report. Then, the sales staff should implement the precise marketing strategy based on the subdivision report of the viewing group to deliver the movie information of BJ Film Company to the appropriate audience timely, accurately, and efficiently. Marketers must use the current Internet platform and information technology to adopt multichannel communication methods to improve marketing performance. Currently, BJ Film Company should not just use the official website for promotional activities regarding promotional channels for the film. The official website has a relatively narrow audience. Under normal circumstances, only viewers who have a high degree of loyalty and recognition to BJ Film Company will often visit the company's official website. However, most moviegoers still obtain movie information through channels such as online recommendation platforms, social circles, and open platforms. Therefore, BJ Film Company must expand its thinking and grasp movie viewing preferences in selecting marketing channels. It should carry out customized channel promotions for different types of moviegoers. The first is the recommendation system. Currently, many network and communication companies in China provide accurate information recommendation services for businesses. BJ Film Company can collect user information according to the way the movie viewer obtains information and use the recommendation system to deliver movie information to them. This can effectively improve the timeliness and effectiveness of viewers' information acquisition. The second is the social circle. With the continuous improvement of networking, many young and middle-aged people prefer to express their views and opinions through social circles. Moreover, social circles have strong dissemination and dependence. Social circle marketing has become an important way to choose current marketing channels. BJ Film Company can use social circle platforms to promote and release films. On the one hand, it can achieve the purpose of accurate publicity. On the other hand, it can quickly spread film information and improve marketing efficiency. In addition, the social circle has a strong writing function, which can provide opinions and suggestions for moviegoers for BJ Film Company. It can also provide timely feedback on moviegoers' opinions through social circles and carry out effective communication and exchanges with moviegoers. Thus, the emotional communication between BJ Film Company and the audience is established, and the audience becomes the main body and participant in the company's film marketing activities.

## 6. Conclusion

The purpose is to allow domestic film companies to obtain accurate marketing and be more profitable. This paper first focuses on the current marketing situation of BJ Film Company. It finds that the company does not have a clear positioning for film prediction, misfunctions in film publicity and distribution, and has a single marketing and promotion method. Secondly, it proposes the necessity and feasibility of applying blockchain technology to the operation and management of film companies. It points out the specific framework and measures and formulates new marketing strategies and safeguard measures for implementing the marketing plan. Finally, three factors affecting the box office: the film's positioning, the film's rating, and the film's marketing, are introduced with 21 variables as input. A film box office's prediction model by RNN is established to predict the premiere monthly box office of the film. The experiment is divided into two parts. The first part predicts the monthly box office of the released films and compares it with the actual box office. Finally, the MAE of the RNN is 7.7%, and the prediction accuracy reaches 92.3%. Thus, the proposed box office prediction model based on RNN has certain accuracy and applicability. The second part predicts the monthly box office of unreleased films and compares it with the target box office. Finally, the predicted monthly box office is 1,451,718.6 CNY, reaching the target monthly box office of 1,414,029.8 CNY. The MAE is only 2.6%, indicating that the proposed marketing strategy has met the marketing needs of the film. Last but not least, this paper has not considered other specific factors affecting the box office, such as release schedule, star effect, and production technology. This will be improved in the follow-up to formulate more scientific marketing strategies for films. The finding has certain reference significance for optimizing the marketing strategy of domestic films.

## Figures and Tables

**Table 1:** Performance of the network.

Performance	Root mean square error (RMSE)	Mean absolute error (MAE)	Accuracy
Result	0.45	0.106	0.80

**Table 2:** Model performance comparison.

Model	RMSE	MAE
The model proposed here	0.010	0.0113
Unimproved LSTM model	0.181	0.1499
ARMA model	0.250	0.216

## Data Availability

The data used to support the findings of this study are included within the article.
